# 170 Collective creativity; promoting physical activity. Outlining a Co-design process for the development of Digital Physical Activity promotion initiatives for Autistic Adults

**DOI:** 10.1093/eurpub/ckae114.049

**Published:** 2024-09-26

**Authors:** Gary Rodgers, Mary Rose Sweeney, Anthony Staines, Natalia Morgulec-Adamowicz, Anna Ogonowska-Słodownik, Debbie Van Biesen, Sean Healy

**Affiliations:** University Of Limerick, Limerick, Ireland; Royal College of Surgeons Ireland, Dublin, Ireland; Dublin City University, Dublin, Ireland; Józef Piłsudski University of Physical Education, Warsaw, Warsaw, Poland; Józef Piłsudski University of Physical Education, Warsaw, Warsaw, Poland; KU Leuven, Leuven, Belgium; University Of Limerick, Limerick, Ireland

## Abstract

**Purpose:**

Autistic students at higher education institutions in Ireland have reported feelings of isolation, depression, and disengagement, all of which contribute to health disparities in this population. It is critical to address these issues, given the recent significant increase in the number of Autistic youths enrolling in higher education. To inform the development of acceptable and effective physical activity promotion programmes, we utilized a co-design process with the aim of co-creating a digital health intervention to promote physical activity among Autistic university students.

**Methods:**

A co-design process [Design Thinking] was utilized for the development of the intervention. First, to empathize with participants, semi-structured interviews were held with Autistic adults. Interviews were recorded, transcribed verbatim, and analysed via reflexive thematic analysis. Actionable intervention targets were defined, and potential solutions ideated. The findings from the interviews informed the exploration and refinement of intervention ideas during co-design workshops. Workshops featured a multidisciplinary development team, including Autistic adults and experts in technology, physical activity promotion, and digital health.

**Results:**

Three central themes were developed during analysis of the interview data: (1) “Psychological Factors” describing participants’ experiences and needs relating to routines, motivation, and self-consciousness/anxiety; (2), “E-Health Ideas and Needs” encapsulated the participants’ preferences and suggestions for digital health intervention components; and (3) “Supportive Spaces” encompassing the participants’ preferences relating to physical and social environments for physical activity. Individual interviews findings informed the ideation of digital health solutions for refinement in co-design workshops; these included interventions focusing on enhancing e-communication about physical activity opportunities on campus, the use of AI Chatbots for physical activity promotion; and the use of online social support groups.

**Conclusions:**

Findings suggest Autistic adults face a variety of interrelated barriers to physical activity. The co-design process enabled the collective design of digital health interventions with the potential to overcome these barriers and support Autistic students to enjoy and more active, engaged University experience.

**Support/Funding Source:**

Erasmus+ Sport grant

